# Culture change and lessons learned from ten years in the VA centers of excellence in primary care education

**DOI:** 10.1186/s12909-024-05390-6

**Published:** 2024-04-26

**Authors:** William G. Weppner, Mamta K. Singh, Joyce E. Wipf, Rebecca Shunk, LeChauncy Woodard, Rebecca Brienza

**Affiliations:** 1 Division of General Internal Medicine, University of Washington School of Medicine, Seattle, WA USA; 2 Primary Care Service, VA Northeast Ohio Health Care System, Case Western Reserve University School of Medicine, Cleveland, OH USA; 3 Division of General Internal Medicine, University of California San Francisco, San Francisco, CA USA; 4 Tilman J. Fertitta Family College of Medicine, Humana Integrated Health System Sciences Institute, University of Houston, Houston, TX USA; 5 Division of General Internal Medicine, Yale School of Medicine, West Haven, CT USA; 6 School of Medicine, University of Washington, Boise VAMC, MSO-111, 500 W. Fort St, 83702; 208.695.0454 Boise, ID USA

**Keywords:** Interprofessional education, Primary care, Collaborative practice, Centers of excellence in primary care education

## Abstract

**Background:**

Team-based care is critical to achieving health care value while maximizing patient outcomes. Few descriptions exist of graduate-level team training interventions and practice models. Experience from the multisite, decade-long Veterans Affairs (VA) Centers of Excellence in Primary Care Education provides lessons for developing internal medicine training experiences in interprofessional clinical learning environments.

**Methods:**

A review of multisite demonstration project transforming traditional silo-model training to interprofessional team-based primary care. Using iterative quality improvement approaches, sites evaluated curricula with learner, faculty and staff feedback. Learner- and patient-level outcomes and organizational culture change were examined using mixed methods, within and across sites. Participants included more than 1600 internal medicine, nurse practitioner, nursing, pharmacy, psychology, social work and physical therapy trainees. This took place in seven academic university-affiliated VA primary care clinics with patient centered medical home design

**Results:**

Each site developed innovative design and curricula using common competencies of shared decision making, sustained relationships, performance improvement and interprofessional collaboration. Educational strategies included integrated didactics, workplace collaboration and reflection. Sites shared implementation best practices and outcomes. Cross-site evaluations of the impacts of these educational strategies indicated improvements in trainee clinical knowledge, team-based approaches to care and interest in primary care careers. Improved patient outcomes were seen in the quality of chronic disease management, reduction in polypharmacy, and reduced emergency department and hospitalizations. Evaluations of the culture of training environments demonstrated incorporation and persistence of interprofessional learning and collaboration.

**Conclusions:**

Aligning education and practice goals with cross-site collaboration created a robust interprofessional learning environment. Improved trainee/staff satisfaction and better patient care metrics supports use of this model to transform ambulatory care training.

**Trial registration:**

This evaluation was categorized as an operation improvement activity by the Office of Academic Affairs based on Veterans Health Administration Handbook 1058.05, in which information generated is used for business operations and quality improvement (Title 38 Code of Federal Regulations Part 16 (38 CFR 16.102(l)). The overall project was subject to administrative oversight rather Human Subjects Institutional Review Board, as such informed consent was waived as part of the project implementation and evaluation.

**Supplementary Information:**

The online version contains supplementary material available at 10.1186/s12909-024-05390-6.

## Background

An interprofessional, team-based model has been shown to improve quality, efficiency, and safety of patient care [1, 2]. This model is essential as health care systems transition to value-based care and is strongly encouraged in undergraduate health profession training by the National Academy of Medicine [3] and American Association of Medical Colleges [4]. Although calls for more support for team-based care have existed for decades [5], successful examples providing meaningful workplace learning environments are limited [3, 6]. 

This manuscript describes the approaches, lessons learned and measured outcomes from a decade-long, multi-site endeavor to transform traditional models of education to interprofessional team-based collaborative care. This manuscript summarizes principles and processes which enabled seven participating Veterans Affairs (VA) programs to successfully develop, implement, evaluate, disseminate and sustain interprofessional models of training. We share specific strategies and professions involved, as well as published trainee, staff, patient and health system outcomes. The lessons learned provide a guidepost to transform other primary care teaching clinics that are interested in engaging more fully in Patient Centered Medical Home practices, and to support graduate training for a more diverse population of interprofessional trainees, from internal medicine, nurse practitioner, pharmacy psychology, and other affiliated health professions.

## Methods

### Setting and participants

The VA health care system transformed its primary care clinics to align with the Patient Centered Medical Home model in 2010 [7]. Subsequent to this transformation, the VA Office of Academic Affiliations (OAA) established “Centers of Excellence in Primary Care Education” (CoEPCE) with the goal of transforming traditional siloed health professional education to collaborative practice models [8]. CoEPCE sites were selected from a competitive application process and received funding to develop and implement programs. Selection was based on innovative proposals to develop models of interprofessional education and team-based care. All sites were located at mid- to large-sized academic primary care clinics. Funding was utilized primarily for salary support for curriculum development, teaching, and evaluation. Each center aimed to design innovative graduate medical education (GME) and health professional training models with sufficient time and structure for all trainees to “learn with, about and from each other” in keeping with National Academy of Medicine recommendations for meaningful interprofessional education and care [3]. Although each site had similar objectives and shared competencies, all were challenged to develop unique models of training, care and culture. Sites were encouraged to share curricula.

The CoEPCE’s were based in academic primary care clinics of initially five, then seven, geographically separate VA institutions with university affiliations. Each site included a variety of unintegrated health professional training programs that were functioning in silos (e.g., internal medicine, nurse practitioner, nursing, pharmacy, psychology, social work, dietetic and physical therapy trainees). As is typical for primary care clinics within the VA, the largest group of trainees were internal medicine residents, both in terms of numbers of trainees and number of years of exposure to the training. New nurse practitioner residencies were created at each site and several established other post-graduate programs, including registered nurse or chiropractic residencies [9]. Trainees were included in evaluations if they provided clinical care in CoEPCE clinic according to their licensure (e.g. graduate health professional trainees), and participated in CoEPCE curricular activities. Trainees typically spent between one and three full academic years in a CoEPCE, depending on the requirements of their training programs. When applicable, perspectives from staff and faculty from the training clinics were included in published qualitative studies. Over the decade of the CoEPCE experiment from 2010 to 2019, more than 1600 trainees were successfully engaged into integrated interprofessional learning and primary care teams for up to three years during training. Local leadership at each site tracked and reported trainee participation at least twice each academic year through a shared reporting portal.

### Program description

#### Foundation

Across the seven sites, there were four educational competencies required at baseline in support of patient-centered care: Shared Decision Making, Sustained Relationships, Performance Improvement and Interprofessional Collaboration [10]. We employed a conceptual model with the foundation that educational transformation needed to align with practice redesign supporting the strong relationship between the patient/caregivers and their primary care team [11]. Sites had different types of participating health professional trainees at a baseline and the predominant training model was within each profession, with very limited shared or overlapping curricula prior to the beginning of the CoEPCE project.

#### Alignment

The initial stage of each program required aligning individual training programs’ accreditation needs, while ensuring meaningful time together in the training model. Attention to overlapping and complementary aspects of different program competencies paralleled discussions of roles and responsibilities in clinic. Particular attention was given to modelling collaborative leadership across professions in curriculum development, implementation, and delivery. During such sessions, interprofessional co-leads purposefully facilitated discussions of profession-specific stereotypes and modelled discussions about how such stereotypes may support or impede collaboration in curricular and clinical care settings. Programs required trainees spend at least 30% of their time in this model to allow sufficient time to interface and develop longitudinal team and patient continuity relationships. This required dedicated time from a core of internal medicine residents, nurse practitioner residents, pharmacy residents, psychology post-doctoral candidates, social work interns and physical therapy trainees. Each participating training program required faculty have dedicated time or teaching commitments to support interprofessional collaboration. This dramatic transformation of profession-specific training models required both commitment and buy-in from each program to participate while still maintaining accreditation requirements. Dedicated faculty time varied depending on overlapping responsibilities, but most participating faculty had at least 10% of their full time equivalent dedicated to the CoEPCE interprofessional work. In addition, travel to conferences was supported by CoEPCE to facilitate dissemination of curricula across sites, typically with participation in 1–2 relevant in-person conferences per year.

#### Approach to curricula

**Fig. 1:**
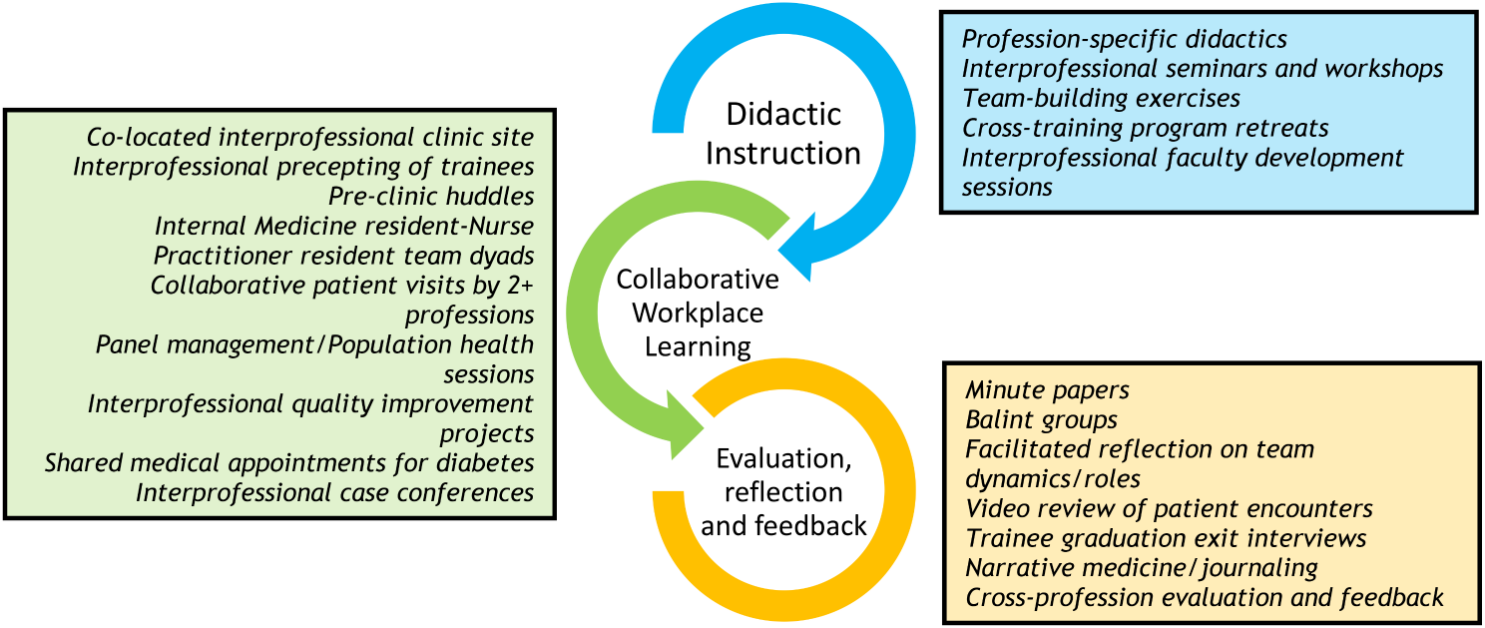
Examples of related curricular innovations spanning three domains of instruction techniques– didactic instruction, collaborative workplace learning, and evaluation/reflection/feedback

To teach core competencies, sites developed curricula using different primary educational modalities: shared workplace learning, didactics and reflection (Fig. 1). These modalities were designed to reinforce and overlap curricula from other areas. One example was an interprofessional case conferences for high-risk/high-need veterans. Initially grounded in didactics related to roles and responsibilities, one site developed weekly team interprofessional case conferences focused on care coordination and team planning for high-utilizing Veterans [12]. The impacts of this conference on trainee and patient outcomes were evaluated with iterative improvements [13, 14]. Through this process, curricula to support the conference as a billable version of clinical care was developed and disseminated to the partner sites [15]. This was not a linear process. Instead, iteration and collaboration across sites led to the next version of the conference, which was successfully implemented at all sites. An immersive, interprofessional workplace learning environment required the VA and the academic affiliates’ commitment to flexible schedules. Most programs instituted an “X + Y” ambulatory block systems for participating internal medicine residencies, with “X” ward weeks alternating with dedicated “Y” ambulatory clinic weeks. New scheduling models and didactic alignment created dedicated time for interprofessional didactics, conferences and projects.

#### Implementation and dissemination

In addition to using the same four core educational competencies, and similar approaches to educational modalities (Fig. 1), sites balanced development of local innovation with efforts to disseminate curricula across sites. Multi-site collaboratives worked to develop curriculum, competencies and quality improvement coursework. Sites employed rapid reflection and change principles to integrate trainee feedback around curricular improvements, team dynamics and roles in real time using practical approaches. Evaluated projects became formal “implementation kits” that each site developed on a yearly basis, based on locally successful curricular innovations. These were shared cross-site via workshops, with academic partners at related educational conferences and via websites more broadly (see Table 1 for examples). Creating sustainable culture change was a conscious effort at all CoEPCE training sites. This required understanding interprofessional stereotypes, noting them, challenging them and discussing some of the underlying truths that served as foundations for how different professions view and interact with each other going forward.

**Table 1:** Examples of curricular projects and related trainee, patient, staff and systems outcomes across center of excellence in primary care education sites

Curriculum (and supporting competencies)	Curricular modality	Professions involved	Trainee outcomes	Patient outcomes	Systems/ Cultural outcomes
Interprofessional seminars on team-based primary care (SDM, SR, IPC)	Didactic instruction; Small group (workshops format); Cross-profession Retreats	IM, NP, PharmD, Health Psych, RN trainees & co-instructors	Enhanced team skills and improved NP students clinical competence [32]	Improved chronic disease management for patients in participating clinics across VA [22]	Participants reported organizational and systemic barriers to changing existing primary care practice [27]
Clinic huddle curriculum (SR, IPC)	Collaborative workplace learning	IM, NP, PharmD, Health Psych, RN trainees & supervisors	Improved understanding and perceived value of team members [33]	Improved chronic disease management for patients in participating clinics across VA [22]	Improved team processes related to clinic flow [33]
Population Health/Panel Management curriculum (IPC, PI)	Didactic instruction, Collaborative workplace learning, Reflection/ feedback/ evaluation	IM, NP, PharmD, RN +/- Psych trainees	Improved confidence and knowledge in management of chronic conditions in patient panel [34]	Improved diabetes care metrics throughout participating clinics across VA [22]	Implementation of curricula and evaluation of learning outcomes across sites [35]
Polypharmacy (SDM, IPC, PI)	Didactic instruction, Collaborative workplace learning, Reflection/ feedback/ evaluation	IM, NP, PharmD, Health Psych, RN trainees	Improvement in trainee knowledge of polypharmacy compared to controls [36]	Medications were appropriately discontinued or had decreased dose and/or frequency in participating Veterans [36]	Trainees perceived that the experience changed their practice in other clinical setting with impacts across system [36]
Interprofessional Quality Improvement projects (IPC, PI)	Didactic instruction, Collaborative workplace learning, Reflection/ feedback/ evaluation	IM, NP, PharmD, Health Psych, RN trainees	Evidence of collaboration and participation by trainees from multiple professions in quality improvement projects [37]	Improved adherence to clinical guidelines and changed opioid prescribing practices in more than one third of assessed patients [38]	Evidence of collaboration and participation by multiple professions in quality improvement projects [37]
Interprofessional Case Conference for High Risk/High Need Patients (SDM, SR, IPC)	Collaborative workplace learning; Small group (case conference) format	IM, NP, PharmD, Health Psych, RN trainees & supervisors	Improved understanding of team roles, increased referral to team members and collaboration [13]	Decreased ER visits and hospitalizations for patients compared to controls [14]	Conference model successfully disseminated across participating CoEPCE sites [15]

Supporting Competencies: SDM = shared decision making, SR = sustained relationships, PI = performance improvement, IPC = interprofessional collaboration

Trainees involved: IM = Internal medicine residents, NP = Nurse practitioner residents, PharmD = Ambulatory pharmacy residents, RN = Registered nurse residents and students, Psych trainees = Psychology interns and post-doctoral candidates

### Program evaluation

The evaluation of the CoEPCE was guided by the Interprofessional Learning Continuum (IPLC) model [3]. This comprehensive approach suggests that effective interprofessional education spans from pre-graduate education through graduate collaborative practice training settings and into professional practice with impacts on the culture of a health care organization. This model encouraged us to move beyond more typical measures of trainee satisfaction and assess trainees’ knowledge and skills, impacts on patient and systems outcomes, and overall culture change. Teaching learners to work in and lead teams was emphasized, with the goal of promoting interest in primary care careers.

Practical evaluation of individual site’s curricula came in the form of real-time, quality-improvement “just-in-time” evaluations supporting an iterative approach to improve content and delivery. Common examples included “Minute Papers,” [16] pre-/post applications of the “Quality Improvement Knowledge Application Tool,” [17] and learner-driven educational portfolios [18]. Sites collaborated to develop new instruments to evaluate curricular implementation, such as population health training and teamwork competencies [19]. Qualitative methods were used to collect perspectives of trainees and exit interviews of graduates related to specific teaching elements. Cross-profession interviewers were used to summarize recommendations to different programs prior to the next academic year.

The multisite evaluation examined a broad spectrum of trainee, faculty/staff, patient and systems outcomes. Multisite evaluations were more in keeping with medical education research, in which *a priori* hypotheses were tested by external evaluators employing methodologically rigorous qualitative and quantitative research techniques. Trainee experiences were collected with repeated cross sectional learner surveys based on a standardized tool developed for VA trainees [20]. These were collected regularly as part of all-site progress reports to monitor success and goal achievement [21]. Clinical outcomes were collected using the VA’s Corporate Data Warehouse, focusing on chronic disease metrics such as diabetes, use of high-risk medication combinations, and appropriate health care utilization patterns [22]. CoEPCE centers received a waiver of informed consent with exemptions, as the evaluation was categorized as an operation improvement activity by the Office of Academic Affairs based on Veterans Health Administration Handbook 1058.05. A comprehensive list of outcomes from interventions across sites of didactic instruction, collaborative workplace learning and reflection/feedback can be found in Appendix 1.

## Results

For trainee and faculty/staff level outcomes, those people involved expressed a positive experience across different settings (see Appendix 1– Participant Outcomes). Notably, trainees who graduated from CoECPE sites indicated overall trainee satisfaction and desire to continue to work in interprofessional collaborative environments, and the desire to serve as change agents to bring interprofessional models to new workplaces upon graduation, based on their CoEPCE experience [23]. Given the traditionally low percentage of internal medicine physicians entering primary care, it was interesting to see the number of participating medicine residents choosing primary care careers after graduation was high. Across sites, 47–81% of internal medicine residents entered a primary care position following graduation [24]. One site indicated a two-fold increase of residents entering primary care, from 36% of historical controls to 75% of CoEPCE graduates [25]. Given the non-randomized nature of this study, we cannot directly ascribe causality between exposure to the CoEPCE and subsequent choice, versus attracting candidates that had a pre-existing interest in primary care. However, the satisfaction reported by trainees during their training supports a positive influence on subsequent career choices. Staff satisfaction in CoEPCE clinical sites was also high; 90% of those employees reported that trainees positively impact job experience, even though 51% of employees agreed that required tasks exceed available time, a finding that was confirmed in subsequent evaluations about staff support ratios in VA interprofessional academic primary care clinics [26]. Overall, there were high levels of satisfaction, low levels of burnout, and the majority indicated they were more satisfied than at their previous workplaces.

Moving beyond trainees and faculty/staff satisfaction and future career paths, patients saw improved outcomes in important clinical areas impacted by interprofessional teams (see Appendix 1– Patient Outcomes). Data from over 49,000 primary care patients representing 100,000 patient-years of care in CoEPCE clinics compared to controls in non-CoEPCE VA academic clinics demonstrated that patients cared for in interprofessional team training environments had improved chronic disease management, less risky medication combinations and more timely referrals to needed behavioral health resources [22]. These support meaningful outcomes related to the core competencies of interprofessional collaboration and shared decision making. These patients also had a lower risk of emergency department visits and hospitalizations for ambulatory care sensitive conditions compared to controls.

Culture change resulting from sustained interprofessional collaboration manifested in many ways. Key structural and curricular innovations were subsequently adopted into the broader residency programs, including adoption of X + Y clinic schedules, incorporation of elements of population health and interprofessional case conference curricula in pre-GME years, expansion of quality improvement curricula, implementation of health policy curricula, as well as starting interprofessional education within other primary care clinics across the residency (Table 1). Graduates reported the program was successful in creating new norms of flattened team hierarchies, broadening graduates’ understanding of role interaction, and teaching relational skills involving teamwork [27]. An important observation suggested that meaningful culture change takes time - for staff and trainees in these newer interprofessional settings, and that those original sites that had more than 5 years of support were more likely to sustain the culture following the termination of CoEPCE grant funding.

Specific comments by internal medicine leadership from different CoEPCE sites provide insight into the development and implementation of a large multi-site interprofessional educational model [24]:

We found that the most successful collaborative learning activities acknowledge the expertise of multiple professions and are focused on patient-centered clinical care. Successful curriculum requires experimentation and sustained incorporation of trainee feedback.

Workplace learning where the learning is embedded in all aspects of clinical care is critical for buy-in from trainees and for sustained improvements in the interprofessional working environment. There is no need to wait until you have a robust clinical interprofessional team-based environment to add trainees. Trainees can be key drivers of the change. Physicians need to be cognizant of the hierarchy of the environment and role model a flattened hierarchy where all team members have a voice.

[CoEPCE] has changed the way we do health care—clearly enhancing our joy in primary care and simultaneously expanding depth of team-based care and quality of the care we deliver—we couldn’t do it without the whole team!

Qualitative approaches to share the trainees’ perspectives were used at individual sites and with cross-site evaluations. Representative quotes from selected citations are listed here:

Internal Medicine Resident (CoEPCE Site #4): “*I think this is the direction of where health care’s going in this country and if this country is going to continue to administer health care, I believe that I’ll be one of the few practitioners [who] comes straight out of residency saying this is the idea of tomorrow as opposed to the ideas of yesterday.”* [28].

Internal Medicine Resident (CoEPCE Site #2): *“We get the opportunity (in the CoEPCE) to really know the patients, really manage the patients, put in a treatment plan, see it enacted, see the results, because we bring the patients back. We see them in a couple of weeks…in a month. We check labs and results. We call patients at home; we follow-up. My colleagues not in this program don’t have the opportunity to do this.”* [29].

Psychology Fellow (CoEPCE Site #1): *“What was so inspiring was the opportunity to work closely with other professionals and learning to use our professional clinical voice and being able to foster relationships with others…for really complicated patients, touching base with primary care trainees, social work, getting together…to brainstorm and talk through some of the difficult pieces and come up with a solution together.”* [29].

Pharmacy Resident (CoEPCE Site #6): *“One of the biggest concepts I will take away from this entire experience is the idea of psychological safety. As a clinician, it is important to feel that your input matters and having the confidence to voice your opinion or share knowledge with your health care team.”* [29].

Nurse Practitioner Resident (CoEPCE Site #3):*“I was really pushed; I was challenged because I had two faculty members that were always there to support me. I saw patients who were much more complex than I ever saw as a student, and I was able to because of the support I received from the team. In addition to that, I also got training in facilitation, motivational interviewing, and patient-centered care.”* [29].

## Discussion

This decade-long experiment in interprofessional team-based training and collaborative practice resulted in broad improvements in clinical learning environment, patient outcomes and the culture of care at participating institutions. Normalizing the change allowed for sustainment of the culture at the end of formal funding. The majority of sites continued interprofessional curricula and supporting structure in the form of “Centers of Education” that are embedded into the training programs. These models were supported based on their history and successful impacts on learners, staff, trainees and patients. The CoEPCE prepared trainees to work not only in local and partner institutions but more broadly in the health care system where quality metrics and remuneration are tied to value-based, team based care [30]. In addition, most sites have been successful at producing practice-ready health professionals that opt to work at their local institution or serve as agents of change in partner institutions, which has diversified and improved the primary care workforce. Given ongoing concerns regarding primary care workforce shortages, ensuring robust training programs is particularly important [31]. At the same time, the CoEPCE experience indicates that collaboration of all professions in the team is needed to optimize care of outpatients– team-based care is not a means to replace a particular profession or role.

Limitations to our findings include heterogeneity in curricula across sites which may limit applicability in other venues. The scope of this project and multiple parties involved in developing, implementing and evaluating different curricula over the CoEPCE timeframe contributed to the challenges in finding uniformly applicable approaches. We had limited direct metrics or outcomes from the four core competencies, particularly related to sustained relationships between CoEPCE trainees and patients, although collected trainee qualitative data suggest that trainees perceived high levels of continuous relationships with peers, patients and faculty. Lessons learned may not be as applicable to non-VA training sites in the US, or to those outside the US. At the same time, the findings reported in this paper, data from papers referenced, and references included in the associated appendix provide myriad examples on approaches that may be more applicable or successful in differing contexts.

Physician leadership in the CoEPCE summarized these important take-home lessons from this experience: (1) Challenge yourself to move beyond the easy outcomes; Evaluation design should not only include trainee satisfaction, but assess actual behavior change, with resultant improved practice, system change, and a transformed culture. (2) Provide dedicated structure for both interprofessional trainees and faculty to develop and understand shared goals, guiding principles, and roles and scopes of practice; this required time and conscious efforts to facilitate venues for socialization by faculty and trainees from different programs to inform collaboration, value of other professions’ unique skills, roles and culture change. (3) Purposefully model interprofessional co-leadership, self-reflection and practical evaluation approaches in a variety of settings; this was deemed important to develop, demonstrate and reinforce these behaviors in learners. (4) Aim for integration of curriculum by intentionally linking didactic instruction, collaborative workplace learning and reflection/evaluation/feedback. (5) “participants were encouraged by their leadership to embrace disruptive chaos; acknowledging the fact that trying new methods of collaboration and instruction may lead to conflicts or failures, but overtly allowing permission for this would eventually promote learning, innovation and successful, sustained interprofessional training programs.

## Conclusion

Developing a set of common competencies and aligning educationalpractice to support cross-profession collaboration and cross-site implementation can provide robust learning environment for interprofessional learners. When implemented successfully, this may be associated with improved trainee experience, staff satisfaction and patient care. Ongoing work to support and disseminate successful approaches in this area are still very much required.

### Electronic supplementary material

Below is the link to the electronic supplementary material.

Supplementary Material 1

## Data Availability

The evidence generated and/or analysed during the current study are available in the publications listed in the supplementary material. The datasets used and/or analysed during the current study that are not referenced are available from the authors (WGW, RB, JW) on reasonable request.
