# Supplementary material for: Culture change and lessons learned from ten years in the VA centers of excellence in primary care education
Source: BMC Med Educ. 2024 Apr 26;24:457. doi: 10.1186/s12909-024-05390-6 (PMC11047004; doi:10.1186/s12909-024-05390-6)
Supplement: Supplementary file 1 — Supplementary Material 1 [file 12909_2024_5390_MOESM1_ESM.docx]

**Appendix 1 – Extended list of themes, examples and outcomes related to interprofessional collaborative education in the Centers of Excellence in Primary Care Education (CoEPCE)**

| **Theme** | **Specific Example(s)** | **Outcome** | **References/ publications** |  |  |  |  |
| --- | --- | --- | --- | --- | --- | --- | --- |
| **Structural Changes** | | | |  |  |  |  |
| Structural changes | - Created Centers for interprofessional (IP) Education with >5 health professions | - Clinic learning environment expanded IP trainees to > 1600 core trainees in 7 CoEPCE sites over 9 years by 2019 - Transformed training out of professional silos - Core postgraduate trainees achieved 30% total training in Primary Care site (Medicine, Advanced Practice Nursing, Pharmacy, Psychology, other health professions) | 1, 2 |  |  |  |  |
|  | - Established New Adult Nurse Practitioner (NP) interprofessional residencies, 1-year transition to practice | - CoEPCEs developed a competency-based assessment toolQualitative analysis from NP resident interviews: 1) bridging the gap to professional practice; 2) expanded appreciation of other health professionals’ role; 3) commitment to interprofessional teamwork; 4) the necessity of mentorship. - Outcomes: change in knowledge, skills and behavior across 7 competency domains improved significantly over the 12 month residency period; NP resident self-evaluation highly correlated with mentor’s evaluation across all time points. | 3, 4  5 |  |  |  |  |
|  | - Creating cultural change with interprofessional training and collaboration | - Qualitative study interviewed 38 graduates and 19 faculty: Interviewed participants perceived program successful in creating new norms of flattened team hierarchies, broadening understanding of role interaction, and teaching interactional skills involving teamwork. - Participants reported organizational and systemic barriers to changing existing primary care practice. | 6 |  |  |  |  |
| **Team-based Care** | | | |  |  |  |  |
| Team-based Care | - Established Clinic Huddles with trainees and PACT (Patient-Aligned Care Teams) staff | - Short team huddles with trainees and PACT members to review patient scheduled, coordinate care, and identify ways to improve team processes - Faculty reinforce collaborative practice and continuous improvement - In interviews, trainees valued their team members and identified improvements in efficiency and quality of patient care due to team-based approach. - Huddle checklists and scores on the Team Development Measure indicated progress in team processes and relationships as the year progressed. | 7, 8 |  |  |  |  |
|  | - Interprofessional case conferences for high risk patients (termed “PACT-ICU”) | - Collaborative Care conferences: - Improved understanding of different professions’ roles by trainees - Increased referral to collaborating professions withing primary care clinic for PACT ICU patients | 9, 10, 11 |  |  |  |  |
|  | - Homeless Care   Comparison of patient outcomes in Homeless Clinic with trainees versus 3 Homeless Clinics without trainees in 3-yrs 2015-18. | - Adding continuity trainees from five health professions did not adversely impact inpatient and outpatient care utilization. - An organized team-based care approach is beneficial for vulnerable patients, provides a meaningful educational experience, builds health professionals' capabilities to care for vulnerable populations. | 12  13 |  |  |  |  |
| **Curricular Innovations** | | | |  |  |  |  |
| Curricular  Innovations | A. Interprofessional Population Panel Management curriculum:   - 168 Trainees completed 122 evaluations.   B. Population panel Management Interprofessional competences | 1. Population panel management (PPM) curriculum evaluation:  - Trainees overwhelmingly reported increased confidence in using PPM and increased knowledge about managing their patient panel. - Reported improved ability to identify patients who would benefit from multidisciplinary care or IP referral. - Directed content analysis revealed that 50% of trainees viewed team members as important system resources.   B. interprofessional competencies developed for graduated autonomy, uniformity across sites | 14  15 |  |  |  |  |
|  | - Dyads: IP Trainees work in pairs providing clinical care | - Improved Teamwork - Students more confident in clinical skills - Reflection opportunity for team dynamics | 16 |  |  |  |  |
|  | - Interprofessional Polypharmacy and Desprescribing Educational Intervention for Primary Care Trainees | - Quantitative and Qualitative Evaluation demonstrated effective model of post-graduate training in complex medication management and de-prescribing that improves residents’ knowledge and skills (compared to a control group)**;** perceived by residents to influence their practice | 17, 18 |  |  |  |  |
|  | - Teaching cultural competency to address human disparities | - Curriculum focuseds on human similarities rather than differences to reduce health disparities - Discuss race as a social construct and not biologic - How to approach cultural differences with a humanistic lens | 19 |  |  |  |  |
| **Patient Outcomes** | | | |  |  |  |  |
| Clinical outcomes | - Comparison of Resident primary care panel Patient Outcomes of 5 CoEPCE sites versus 5 comparable academic VA non-CoEPCE sites | Compared with resident clinicians who did not participate in the CoEPCE initiative, CoEPCE training was associated with improved clinical outcomes among 49,000 patients on trainee panels: Improved diabetes management as measured by A1c; Improved annual renal testing in patients with diabetes; Fewer older patients receiving high-risk medications; More timely Mental Health referrals; Fewer hospitalizations for ambulatory sensitive conditions | 20 |  |  |  |  |
|  | - High acuity care primary care conferences (termed PACT ICU) impact on outcomes | PACT-ICU conferences: assessment of patients with high acuity (CAN scores): reduced emergency department visits, reduced hospitalizations | 21 |  |  |  |  |
|  | - Improving referral process by integrating Physical Therapy into primary care clinics | - Compared to 2 traditional academic primary care clinics, the clinic with the PT integrated into the primary care clinic resulted in increased placing and completions of PT consults; reduced discontinuations/cancellations of referrals and reduced wait time to see the PT. | 22 |  |  |  |  |
| **Quality Improvement** | | | |  |  |  |  |
| Quality  Improve-  ment  Projects | - Project summaries - Mix of new and continuing projects documented | - Quality improvement projects documented in CoE Coordinating Center semi-annual reports: - Average of 10-15 projects/year per site - Most common themes: HTN, DM, ED Utilization, Cancer screening - 89% multi-professional (Seattle data 2018-19) | 2, 23 |  |  |  |  |
|  | - Survey of primary care faculty and trainees on perceptions of patients’ ability to utilize telemedicine, and their own comfort with use. | - Trainees and staff providers (faculty) rated own skills in telemedicine as high level of comfort - Lower ratings by providers of perceived patient ability to use tools for virtual visits. | 24 |  |  |  |  |
|  | - VA Medical Home: Impact on Diabetes Medication Compliance | - CoEPCE Resident-led project. One-year refill-based medication possession ratios were calculated at the patient level - Elements of VA Patient-Centered Medical Home were associated with greater adherence to oral medications in patients with diabetes, including access to care, having a respectful office staff, and utilization of telephone encounters. | 25 |  |  |  |  |
|  | - Unstructured interviews of 11 patient-aligned primary care teams and 4 QI facilitators and coaches in a large academic medical practice | - Interdisciplinary QI team training requires data collection support and dedicated coaching resources in addition to QI training - QI teams should consider data/informatics person as part of the QI team - Dedicated team time for QI projects ensure success | 26 |  |  |  |  |
| **CoEPCE Participant Outcomes** | | | |  |  |  |  |
| Trainee  Perspective | - Annual CoEPCE Participants Survey administered to all trainees - Assessment of satisfaction with CoEPCE by profession - Alumni data (qualitative data via interviews) | - High level of satisfaction with interprofessional training program overall for all sites - Variation by professions each year | 2, 27 |  |  |  |  |
| Trainee  Graduate  Career  Choice | - Interest in primary care career before and after CoE training - Selection of primary care career choice | - CoEPCE all-site Trainee annual survey 2019:   - All 7 sites: Center respondents self-reported on average that interest in primary care career rose with CoE compared to before training, or preserved high level of interest in primary care.   - 3 sites with Primary Care career choice focus:   - 47-81% Primary Care career choice   - One site tracking career choice for all program graduates:   - 75% of CoEPCE residents selected primary care careers (3-yr cohorts 2011-2019, graduates 2014-2019) compared to 36% of IM program historical controls. | 28, 29 |  |  |  |  |
| Faculty and  Staff satisfaction | - Staff satisfaction survey - Semi-structured individual interviews with RN and clinical and clerical staff (n=32) - Alignment with training mission - Burnout data by OAA to sites | - Efforts to improve interprofessional collaboration among trainees and providers have positive spillover effects for primary care medical home staff members. - Staff members reported that they play and education role for trainees that is not always acknowledged. - High level of CoE faculty and staff alignment with CoE training mission - Improved job satisfaction than pre-CoEPCE - Using 1-item validated burnout question: Reduced burnout CoE faculty/staff than other published PACT data (22% CoE + vs typically >35-50% in primary care | 27, 30, 31, 32 |  |  |  |  |
| **Culture change** | | | |  |  |  |  |
| Adoption/  Dissemination to Academic Affiliations | - Interprofessional Faculty Development and co- precepting and teaching | - Culture change with sustained active Interprofessional Faculty develop program - 3-fold increase in CoE academic affiliations from 11 in 2011 to 33 by 2016 (Schools of Medicine and Nursing) - Impact on academic affiliate | 2, 33 |  |  |  |  |
|  | - Implementation Kits online | - Seattle CoEPCE Implementation Kits:   - Population panel management Implementation Kit (active since 2018); Detailed curricular elements, session examples with video clips, curriculum evaluation, competencies.  - Shared Decision-Making Implementation Kit active since in 2021   - Boise CoEPCE: PACT-ICU Implementation Kit (active since 2017); Curriculum, other - San Francisco CoEPCE Implementation Kits. Development of a Interprofessional collaborative Case Conference. Team Retreats for Interprofessional Trainees/Staff. QI in Primary Care Through a Longitudinal, Project-Based, Interprofessional Curriculum. Huddle-coaching in primary care for trainees and staff to support team-based care. | 34  35, 36, 37, 38 |  |  |  |  |
|  | - CoE Primary Care Novel rotations for Medicine Residents | - Eighty-five of 102 (83%) possible novel rotation evaluations from 2014 to 2017 were reviewed (evaluations available) - Residents reported that CoEPCE rotations had a positive effect on their care of patients, career choice, and opportunities to interface with faculty role models. - Internal Residency adopted select rotations for all primary care track and CoE rotation electives to all residents (ie homeless care, clinician-educator block) - Similar rotations developed for Nurse Practitioner residents and for NP students | 29 |  |  |  |  |
|  | - Development of Professional Identity and formation of Teams in CoEs | - Assess effect of an interprofessional training program on professional identify and team development - Initially IM residents struggled to undertand NP’s roles and responsibilities, whereas NP residents doubted their ability to work alongside physician residents. At the end of the academic year, these uncertainties disappeared, and the two unique groups became a team that realized the importance of relationships, growth, and collaboration both in the clinic and the classroom. - MD and NP Residents found a way to marry divergent philosophical models, and actively worked together to establish a transformed culture with patient care excellence | 39 |  |  |  |  |

References in Appendix Table:

1. Gilman SC, Chokshi DA, Bowen JL, Rugen KW, Cox M. Connecting the dots: interprofessional health education and delivery system redesign at the Veterans Health Administration. Academic Medicine. 2014; 89(8):1113-1116.

2. Harada ND, Traylor L, Rugen KW, et al. Interprofessional transformation of clinical education: The first six years of the Veterans Affairs Centers of Excellence in Primary Care Education, Journal of Interprofessional Care, 2018; 20:1-9.

3. Rugen KW, Watts SA, Janson S, et al. Veteran Affairs Centers of Excellence in Primary Care Education: Transforming nurse practitioner education, Nursing Outlook. 2014; 62(2):78-88.

4. Zapatka S, Conelius J, Edwards J, Meyer E, Brienza RS. Pioneering a Primary Care Adult Nurse Practitioner Interprofessional Fellowship. Journal of Nurse Practitioners. 2014 June; 10(6):378-386.

5. Rugen KW, Dolansky MA, Dulay M, King S, Harada N. Evaluation of Veterans Affairs primary care nurse practitioner residency: Achievement of competencies. Nursing Outlook,2018; 66(1): 25-34.

6. Hulen E, Edwards ST, Poppe AP, Singh MK, Shunk R, Tuepker A. [Creating change, challenging structure: graduate and faculty perspectives on the implementation of an interprofessional education program in veterans’ affairs primary care.](https://pubmed-ncbi-nlm-nih-gov.offcampus.lib.washington.edu/31696759/) Journal of Interprofessional Care. 2020; 34(6):756-762.

7. [Shunk](https://pubmed-ncbi-nlm-nih-gov.offcampus.lib.washington.edu/?term=Shunk+R&cauthor_id=24362383) R, [Dulay](https://pubmed-ncbi-nlm-nih-gov.offcampus.lib.washington.edu/?term=Dulay+M&cauthor_id=24362383) M, [Chou](https://pubmed-ncbi-nlm-nih-gov.offcampus.lib.washington.edu/?term=Chou+CL&cauthor_id=24362383) CL, [Janson](https://pubmed-ncbi-nlm-nih-gov.offcampus.lib.washington.edu/?term=Janson+S&cauthor_id=24362383) S, [O'Brien](https://pubmed-ncbi-nlm-nih-gov.offcampus.lib.washington.edu/?term=O%27Brien+BC&cauthor_id=24362383) BC. Huddle-coaching: dynamic intervention for trainees

and staff to support team-based care. Academic Med 2014; 89(2):244-250.

8. Gardner AL, Shunk R, Dulay M, Strewler A, O'Brien B. [Huddling for High-Performing Teams.](https://pubmed-ncbi-nlm-nih-gov.offcampus.lib.washington.edu/30766382/) Federal Practitioner. 2018; 35(9):16-22.

9. Sordahl J, King IC, Davis K, et al. Interprofessional case conference: impact on learner outcomes, Translational Behavioral Medicine. 2018; 8(6):927-931.

10. Weppner WG, Davis K, Sordahl J, et al. PACT ICU – Interprofessional Care Conferences for High-Risk Primary Care Patients. Federal Practitioner. 2018; 35(12):34-41.

11. O’Brien BC, Patel SR, Pearson M, et al. Twelve tips for delivering successful interprofessional case conferences. Medical Teacher*.* 2017;39(12):1214-1220.

12. Gelberg L, Edwards ST, Hooker ER, Niederhausen M, Shaner A, Cowan B, Warde C. Integrating Interprofessional Trainees into a Complex Care Program for Homeless Veterans: Effects on Health Services Utilization. J Gen Intern Med. 2021; 36(12):3659-3664.

13. Warde C, Soh M, Stuber M, Tilden V, Gelberg L. An Interprofessional Team Performance Training Program for the Care for Vulnerable Populations in the Ambulatory Setting. J of Interprofessional Education and Practice 2020; 20:1-6.

14. Kaminetzky CP, Beste LA, Poppe AP, et al. Implementation of a novel population panel management curriculum among interprofessional health care trainees. BMC Medical Education, 2017:*17*(1), 264-270.

15. Dulay M, Bowen JL, Weppner WG, et al. Interprofessional population health advocacy: Developing and implementing a panel management curriculum in five VA primary care practices. Journal of Interprofessional Care. 2018; 10:1-11.

16. Gardner AL, Clementz L, et al. The Dyad Model for Interprofessional Academic Patient Aligned Care Teams. Federal Practitioner. 2019; 36(2):88-93.

17. Mecca MC, Thomas JM, Niehoff KM, et al. Assessing an Interprofessional Polypharmacy and

Desprescribing Educational Intervention for Primary Care Post-Graduate Trainees: A Quantitative and

Qualitative Evaluation. J Gen Intern Med. 2019; 34 (7): 1220-1227.

18. Thomas JM, Mecca MC, Niehoff KM, et al. Development and Validation of a Polypharmacy Knowledge Assessment Instrument. American Journal of Pharmaceutical Education. 2019; 83(5):6435.

19. Clementz L, McNamara M, Burt NB, Sparks M, Singh MK.  Starting With Lucy: Focusing on Human Similarities Rather Than Differences to Address Health Care Disparities, Academic Medicine. 2017; 92(9): 1259-1263.

20. Edwards ST, Hooker ER, Brienza R, et al. Association of a Multisite Interprofessional Education Initiative with Quality of Primary Care. JAMA Network Open. 2019; 2(11):e1915943.

21. Weppner WG, Davis K, Tivis R, Willis J, Fisher A, King I, Smith CS. Impact of a complex chronic care patient case conference on quality and utilization. Translational Behavioral Medicine. 2018; 8(3) 366-34.

22. McKay S, Buono F, Walker J, Glinski Carly, Printz D, Brienza RS. Impact of Interprofessional Embedding of Physical Therapy in a Primary Care Training Clinic. Journal of Interprofessional Care, 2021; 35(4):532-537.

23. Hunt LM, Fisher AK, King IC, Wilper AP, Speroff E, Weppner WG. Primary care collaborative practice

in quality improvement: A description of an interprofessional curriculum; American Journal of Health-

System Pharmacy. 2018; 75(21):1729-1735.

24. Samples LS, Martinez J, Beru YN, Rochester MR, Geyer JR. Provider Perceptions of Telemedicine

Video Visits to home in a Veteran Population. Telemedicine J E Health. 2021 Apr;27(4):422-426.

25. Meo N, Wong E, Sun H, Curtis I, Batten A, Fihn SD, Nelson K. Elements of the Veterans Health

Administration Patient-Centered Medical Home are Associated with Greater Adherence to Oral

Hypoglycemic Agents in Patients with Diabetes. Population Health Management. 2018;21(2):116-122.

26. Watts B, Lawrence RH, Singh S, Wagner C, Augustine S, Singh MK. Implementation of quality improvement skills by primary care teams: case study of a large academic practice. J Prim Care Community Health. 2014 Apr 1;5(2):101-6.

27. Hulen E, Edwards ST, Poppe AP, Singh MK, Shunk R, Tuepker A. Creating change, challenging structure: graduate and faculty perspectives on the implementation of an interprofessional education program in Veterans Affairs primary care. J Interprofessional Care. 2020; 34(6):756-762.

28. Wipf JE, Weppner WG, Brienza RS, Singh MK. Interprofessional team-based training increases medicine resident primary care career choice: a decade of experience with a novel education program model. American Medical Association-sponsored GME Innovations Virtual Summit Video Abstract, Oct 5-7, 2020.

29. Deeds S, Wipf JE, Corning K, Takahashi T. Expanding Primary Care Experiences with novel rotations for Residents at One University-affiliated Veteran Affairs Center of Excellence in Primary Care Education. J Graduate Medical Education.  *2019*; 11(6):691-697.

30. Hulen E, Edwards ST, Poppe AP, Singh MK, Shunk R, Tuepker A. Creating change, challenging

structure: graduate and faculty perspectives on the implementation of an interprofessional education

program in veterans’ affairs primary care. J Interprofessional Care. 2019; 7:1-7.

31. Newell S, O'Brien B, Brienza R, et al. Experiences of Patient-Centered Medical Home Staff Team Members Working in Interprofessional Training Environments. J Gen Intern Med. 2020;35(10):2976-2982.

32. Harada ND, Rajashekara S, Sansgiry S, Rugen KW, King S, Gilman SC, Davila JA. Developing Interprofessional Primary Care Teams: Alumni Evaluation of the Department of Veterans Affairs Centers of Excellence in Primary Care Education Program. Journal of Med Ed and Curr Dev. 2019; 6:1-14.

33. King, I., Strewler, A., Wipf, J., Singh, M., Painter, E., Shunk, B., Brienza, R., Tivis, R., Weppner, W., Davidson, M., Willis, J., Gordon, J., Smith, C. Translating innovation: Exploring dissemination of a unique case conference. Journal of Interprofessional Education and Research. 2017; 6:55-60.

34. Geyer J, Rao M, Golob A, Wipf JE. Population panel management Implementation Kit online. UW Website URL: <https://depts.washington.edu/vapcc/>; accessed 8/29/2023

35. Boise VA Center of Educcaiton for Interprofessional Collaboration. PACT ICU Implementation kit - <https://boisevacoe.org/>; accessed 8/29/2023

36. Pearson PM, O’Brien OB, Shunk RL. Development and Evaluation of an Interprofessional Collaborative Case Conference Series in Primary Care. MedEdPORTAL. 2014; 10:9937.

37. Patel S, [O'Brien BC](https://profiles.ucsf.edu/bridget.obrien), [Dulay M](https://profiles.ucsf.edu/maya.dulay), Earnest G, **S**hunk RL. Team Retreats for Interprofessional Trainees and Clinic Staff: Accelerating the Development of High-Functioning Teams. MedEdPORTAL. 2018; 14:10786.

38. [Dulay M](https://profiles.ucsf.edu/maya.dulay), [Saxe JM](https://profiles.ucsf.edu/joanne.saxe), Odden K, Strewler A, Lau A, [O'Brien B](https://profiles.ucsf.edu/bridget.obrien), Shunk R. Promoting Quality Improvement in Primary Care Through a Longitudinal, Project-Based, Interprofessional Curriculum. MedEdPORTAL. 2020; 16:10932.

39. Weppner WG, Fisher A, Hagman M, et al. Taking SGIM's Teaching Educators Across the Continuum of Healthcare on the Road - A Local, Interprofessional Faculty Development Innovation; J Graduate Medical Education. 2017, 9(3): 378-379.
